# Lung Ultrasound Diagnostic Accuracy in Neonatal Pneumothorax

**DOI:** 10.1155/2016/6515069

**Published:** 2016-05-03

**Authors:** Luigi Cattarossi, Roberto Copetti, Giacomo Brusa, Stefano Pintaldi

**Affiliations:** ^1^Department of Neonatology, Santa Maria della Misericordia University Hospital, 33100 Udine, Italy; ^2^Emergency Department, Latisana General Hospital, 33053 Latisana, Italy; ^3^Department of Pediatrics and Neonatology, Ospedali Riuniti Marche Nord, Pesaro, Italy

## Abstract

*Background*. Pneumothorax (PTX) still remains a common cause of morbidity in critically ill and ventilated neonates. At the present time, lung ultrasound (LUS) is not included in the diagnostic work-up of PTX in newborns despite of excellent evidence of reliability in adults. The aim of this study was to compare LUS, chest X-ray (CXR), and chest transillumination (CTR) for PTX diagnosis in a group of neonates in which the presence of air in the pleural space was confirmed.* Methods*. In a 36-month period, 49 neonates with respiratory distress were enrolled in the study. Twenty-three had PTX requiring aspiration or chest drainage (birth weight 2120 ± 1640 grams; gestational age = 36 ± 5 weeks), and 26 were suffering from respiratory distress without PTX (birth weight 2120 ± 1640 grams; gestational age = 34 ± 5 weeks). Both groups had done LUS, CTR, and CXR.* Results*. LUS was consistent with PTX in all 23 patients requiring chest aspiration. In this group, CXR did not detect PTX in one patient while CTR did not detect it in 3 patients. Sensitivity and specificity in diagnosing PTX were therefore 1 for LUS, 0.96 and 1 for CXR, and 0.87 and 0.96 for CTR.* Conclusions*. Our results confirm that also in newborns LUS is at least as accurate as CXR in the diagnosis of PTX while CTR has a lower accuracy.

## 1. Introduction

Pneumothorax (PTX) still remains a common cause of morbidity in critically ill and ventilated neonates despite the increasing use of antenatal corticosteroids, surfactant, and less aggressive ventilation. PTX during respiratory distress is associated with increased risk of intraventricular hemorrhage, chronic lung disease, and death [1]. Symptomatic PTX occurs in 0.08% of all live births and in 5.00% to 7.00% of all infants with birth weight <1500 g [2]. With respect to gestational age, the overall rate of PTX according to maturity is 4.0%, 2.6%, and 6.7%, respectively, in early preterm, moderate-late preterm, and term neonates [3].

In a newborn, the diagnostic work-up consists mainly of chest X-rays (CXR) and chest transillumination (CTR). In adults, LUS demonstrated higher sensitivity to diagnosing PTX when compared to supine CXR, and the excellent performance of ultrasonography supported the routine use of this technique for the detection of pneumothorax [4–10].

To our knowledge, there is no paper on this topic in neonatal age but only few case reports [11].

The aim of this study was to compare LUS, chest X-ray (CXR), and chest transillumination (CTR) for PTX diagnosis confirmed by chest aspiration or chest tube insertion.

## 2. Ultrasound Signs of Pneumothorax

**Figure 1:**
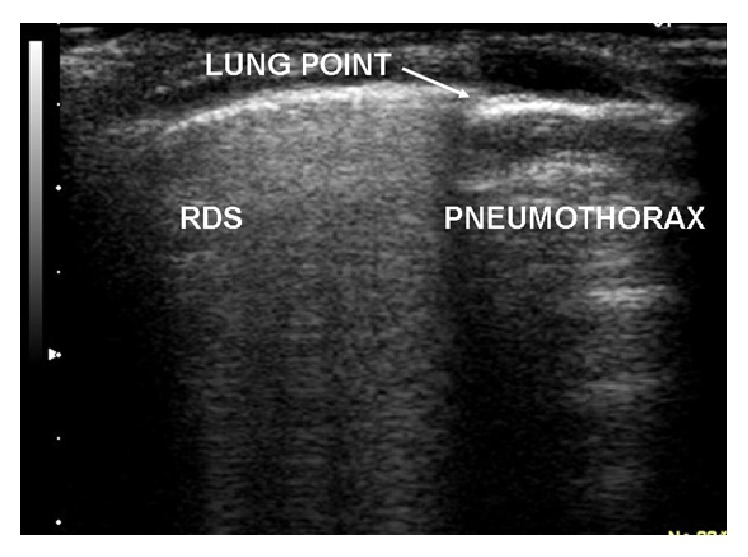
Static image of lung point (arrow) in an infant suffering from RDS. Note the coalescent B lines in the left side of the image (sign of RDS); they suddenly disappear at the edge of PTX (lung point).

Four sonographic signs are useful in ruling in and ruling out PTX:

1. Absence of lung sliding (video #1, in Supplementary Material available online at http://dx.doi.org/10.1155/2016/6515069).
2. Absence of B lines (Figure 1).
3. Absence of lung pulse.
4. Presence of lung point (Figure 1 and video #2).

Patients should be in a reclined position during investigation, because air collects within the anterior nondependent portions of the pleural space [5].

Lung sliding represents a regular rhythmic movement synchronized with respiration that occurs between the parietal and visceral pleura that are both in direct apposition. Lung sliding is not observable at the moment in which air is stratified between the two pleural layers. For the same reason B lines are absent, with these vertical artifacts arising from the visceral pleura.

In the PTX, especially on the left, the transmission of cardiac systoles on the pleural line, the so-called “lung pulse,” is lost.

The lung point refers to the depiction of the typical pattern of PTX. It is the absence of any lung sliding adjacent to an area of sliding, which represents the physical limit of pneumothorax. In massive PTX, lung points are obviously absent.

In adults, ultrasound has demonstrated superior sensitivity and similar specificity with respect to standard radiography in the diagnosis of PTX and recent studies showed that there is a good correlation between the location of “lung point” and the volume of PTX evaluated with CT scan, with LUS being more accurate than CXR in this respect [8, 9].

## 3. Study Design

In adults, the gold standard for PTX diagnosis is CT scan [5, 7]. Obviously, this criterion cannot be applied in a neonatal context. Therefore, to base our result on a reliable gold standard we decided to enroll in the study patients with PTX confirmed by aspiration, thus with clear evidence of PTX.

The study was done at the Neonatal Intensive Care Unit of Santa Maria della Misericordia Hospital (Udine, Italy), a tertiary University Hospital with 10 beds for intensive care and 20 beds for intermediate care.

Newborns admitted in the Neonatal Intensive Care Unit presenting respiratory distress were eligible for the study. LUS, CTR, and CXR were done at the time of recruitment. Neonates with lung point at or beyond the anterior axillary line underwent chest aspiration or chest drainage according to the evidence that in adults lateral extension of lung point correlates with a large PTX [8, 9]. The control group was composed of neonates presenting respiratory distress but without LUS consistent with PTX.

LUS is routinely utilized in our unit and is part of the work-up of neonates with respiratory distress. The majority of the medical staff is trained in LUS. The operator was aware of clinical state of the patient.

## 4. Patients and Methods

From January 1st 2012 to December 31st 2014, 23 neonates with PTX who required aspiration or chest drainage were enrolled (Table 1). All infants had CXR, CTR, and LUS. Gestational age ranged from 29 to 41 weeks (mean ± SD: 36 ± 5), with weight from 998 to 3950 grams (mean ± SD: 2120 ± 1640). Fourteen were born by cesarean section. All of them suffered from respiratory distress (Transient Tachypnea of the Newborn 9, Respiratory Distress Syndrome 6, Meconium Aspiration Syndrome 3, Spontaneous PTX 3, Hydrops 1, and Chylothorax 1) and showed clinical signs suggestive of PTX (worsened respiratory distress, sudden deterioration of oxygenation, and need of more sustained respiratory support).

PTX was seen between 4 and 72 hours of life (mean ± SD: 28 ± 18 hours). None had hemodynamic instability.

Six infants were supported with conventional mechanical ventilation, 1 was supported with High Frequency Oscillatory Ventilation (HFOV), 11 were supported with Continuous Positive Airway Pressure (CPAP) through nasal prongs, and 5 were supported with Infant Flow CPAP. All of them required pleural aspiration: in 5 neonates, needle aspiration was performed and in 18 neonates a chest drainage was positioned connected to a sealed aspiration system.

The control group consisted of 26 neonates admitted on the same time frame of the cases, suffering from respiratory distress (Transient Tachypnea of the Newborn 14, Respiratory Distress Syndrome 9, and Meconium Aspiration Syndrome 3), but without PTX (Table 1). Gestational age ranged from 26 to 42 weeks (mean ± SD: 34 ± 4); weight ranged from 744 to 4706 grams (mean ± SD: 2252 ± 990). Five infants were supported with conventional mechanical ventilation, 2 with High Frequency Oscillatory Ventilation (HFOV), 12 with Continuous Positive Airway Pressure (CPAP) through nasal prongs, and 7 with Infant Flow CPAP.

All infants had an anteroposterior CXR in supine position. CTR was performed with a Fiber Optic Light (Storz Cold Light Fountain 488B, Karl Storz, Tuttlingen, Germany) in a darkened area according to the standard modalities. LUS was done using a Prosound *α*7 Ultrasound System (Hitachi-Aloka Medical, Stuttgart, Germany) and a high frequency linear probe (13 MHz). Five different neonatologists trained in chest sonography were in charge of LUS execution.

## 5. Statistical Analysis

Continuous variables are reported as means ± SD and were compared by using a Wilcoxon test (SPSS version 14.0 statistical software, IBM Corporation, Armonk, NY). The significance was set at *p* < 0.05 for each variable.

PTX was diagnosed (positive condition) by the presence of air in the pleural space confirmed at chest aspiration. In controls, PTX was excluded (negative condition) when LUS, CTR, and CXR were negative. Sensitivity, specificity, PPV, NPV, and 95% confidence interval (CI) were calculated by standard formulas.

## 6. Results

All four LUS signs of PTX were seen in the 23 patients. CXR did not detect PTX in one patient; CTR was negative in 3 cases. The patient with negative CXR had also a negative CTR. The lung point has been seen in the anterior axillary line in 18 cases and in the middle axillary line in the remaining 5. In the infant with PTX and CXR and CTR negative as well in the two patients with CTR negative, LUS showed the lung point at the anterior axillary line. None of the infants had pleural effusion either to CXR or to LUS.

In the control group, none had LUS or CXR positive for PTX; in one infant CTR was judged positive. Sensitivity and specificity in diagnosing PTX were therefore 1 for LUS, 0.96 and 1 for CXR, and 0.87 and 0.96 for CTR. Positive predictive value was 1 for LUS and CXR and 0.96 for CTR. Negative predictive value was 1 for LUS, 0.96 for CXR, and 0.88 for CTR.

Table 2 summarizes the results in terms of sensitivity and specificity and positive and negative predictive value of LUS, CXR, and CTR.

## 7. Discussion

In our study, LUS showed absolute sensitivity and specificity referring to the gold standard represented by air aspiration from the pleural space. CT scan which is the gold standard for imaging PTX diagnosis [5, 8] is not feasible in neonates for practical reasons and exposure to ionised radiation.

At the present time, LUS has demonstrated high sensitivity and specificity in RDS and TTN diagnosis [12] and it is well correlating with oxygenation status [13], but it is not included in the diagnostic work-up of PTX in neonates. So far, only case reports have been reported on this topic in neonatal age. Differently, in adults LUS is accepted as reliable and accurate diagnostic tool and has demonstrated superior sensitivity and similar specificity with respect to standard radiography in the diagnosis of pneumothorax [10].

The anatomical features of neonates (thinner chest wall, smaller thoracic width, and lung mass) facilitate LUS imaging. Moreover, the underlying lung diseases of the neonates that develop PTX generate constantly the presence of B lines. Their absence is therefore unusual in these conditions and makes the detection of lung point easier. Our results confirm that also in newborns LUS is at least as accurate as CXR in the diagnosis of PTX. On the other hand, PTX is often a respiratory emergency and requires a rapid initiation of therapeutic intervention. The delay required in obtaining CXR may be life threatening. LUS can be performed rapidly at bedside without delay in the diagnosis and treatment.

Lung point establishes the extension of the PTX and may be utilized in differentiating between small and large PTX. In our limited case series, larger PTX at CXR had a lung point situated at middle axillary line. We speculate that a lung point at or beyond the middle axillary line is consistent with a large PTX.

It is known that CTR allows rapid detection of PTX at bedside. However, it is also well known that its limits are due to the possibility of false positive (infants with chest wall edema, subcutaneous chest wall air, pneumomediastinum, or severe pulmonary interstitial emphysema) or negative (thick chest wall, darkly pigmented skin, or nonadequate light conditions). Also our study confirms that CTR is less accurate than CXR and LUS.

LUS allows according to the level of lung point avoiding mistakes in chest puncture. In our series, all the procedures have been performed under ultrasound assistance without complications. Lung expansion can be followed by LUS monitoring lung point disappearance. LUS is an excellent tool to evidence the recurrence of PTX without exposure to ionising radiation.

We acknowledged some limitations to our study. The patient number is small, but we based our results on a reliable gold standard, and our choice explains the limited patient sample.

The operators who performed LUS were not blinded to clinical data and neonates appearance so the excellent results of ultrasound may be conditioned by this fact; on the other hand we need tools that are accurate when integrated with the clinical information at bedside. Finally, ultrasound is affected by the operator experience. The neonatologists involved in the study were trained in LUS technique and data could be worse for novice. However, it was demonstrated that LUS learning curve is quite short [14] and even beginner operators had high reliability in neonatal diagnosis when compared with experienced one [15].

This is the first study that evaluates the sensitivity and specificity of LUS in diagnosing neonatal PTX, and more data with larger samples are needed to confirm this good performance.

## 8. Conclusion

Our study demonstrated that also in newborns LUS had optimal sensitivity and specificity in diagnosing PTX. The lung point site, as demonstrated in adults, may be useful to distinguish between large and small PTX.

Therefore, we retain that LUS should be included in diagnostic work-up when PTX is suspected in a neonate.

## Supplementary Material

Clip # 1: Note the absence of lung sliding due to the presence of PTX.
Clip # 2: Note the lung point in the middle of the screen.

## Figures and Tables

**Table 1:** Demographic data and type of respiratory support.

	PTX	Control	*p*
Gestational age (mean ± SD)	36 ± 5	34 ± 4	ns
Birth weight (mean ± SD)	2120 ± 1640	2252 ± 990	ns
Male/female	17/6	16/10	ns
Apgar score at 1 minute (mean ± SD)	4 ± 4	6 ± 2	ns
Apgar score at 5 minutes (mean ± SD)	7 ± 1	8 ± 2	ns
Vaginal delivery∖cesarean section	9/14	8/15	ns
Deaths	1	1	ns
CPAP with nasal prongs	11	12	ns
CPAP or BiPAP with Infant Flow	5	7	ns
Conventional mechanical ventilation	6	5	ns
HFOV	1	2	ns

**Table 2:** Sensitivity and specificity and positive and negative predictive value of LUS, CXR, and CTR.

	LUS	CXR	CTR
Sn (IC 95%)	1.00 (1.00-1.00)	0.96 (0.87–1.00)	0.87 (0.73–1.00)
Sp (IC 95%)	1.00 (1.00-1.00)	1.00 (1.00-1.00)	0.96 (0.87–1.00)
VPP (IC 95%)	1.00 (1.00-1.00)	1.00 (1.00-1.00)	0.95 (0.86–1.00)
VPN (IC 95%)	1.00 (1.00-1.00)	0.96 (0.88–1.00)	0.88 (0.75–1.00)

## Abbreviations

CXR: Chest X-ray
CTR: Chest transillumination
CPAP: Continuous Positive Airway Pressure
HFOV: High Frequency Oscillatory Ventilation
LUS: Lung ultrasound
PTX: Pneumothorax.

## Additional Points

What is known on this subject is that lung ultrasound has demonstrated to be as much reliable as chest X-ray in detecting pneumothorax in adults. At the present time, this possibility has not been explored in neonates. What this study adds is that it shows that lung ultrasound compared with chest X-ray and chest transillumination had at least the same reliability of chest X-ray and was more accurate than chest transillumination in diagnosing pneumothorax.
